# Editorial: Extracellular vesicles as potent modulators of immunity

**DOI:** 10.3389/fcell.2023.1278498

**Published:** 2023-08-28

**Authors:** Krzysztof Bryniarski, Lola Fernández-Messina, Philip W. Askenase, Katarzyna Nazimek

**Affiliations:** ^1^ Department of Immunology, Jagiellonian University Medical College, Krakow, Poland; ^2^ Department of Cell Biology, Faculty of Biological Sciences, Complutense University of Madrid, Madrid, Spain; ^3^ Immunology Unit from Hospital Universitario de La Princesa, Universidad Autónoma de Madrid and Instituto de Investigación Sanitaria La Princesa (IIS-IP), Madrid, Spain; ^4^ Intercellular Communication in the Inflammatory Response, Vascular Pathophysiology Area, National Center for Cardiovascular Research (CNIC), Madrid, Spain; ^5^ Section of Rheumatology, Allergy and Clinical Immunology, Department of Internal Medicine, Yale University School of Medicine, New Haven, CT, United States

**Keywords:** allergy, autoimmunity, biotherapeutics, cancer, exosomes, extracellular vesicles, immunomodulation

For over 20 years, the research on membranous nanoparticles produced and released extracellularly by virtually all cells of living organisms has been developing more and more intensively, resembling a snowball rolling down the slope. In 2011, the term extracellular vesicles (EVs) was introduced into common use, defining all cell-secreted particles with a bilayer lipid membrane, and efforts were made to standardize the nomenclature of EV’s subpopulations, as well as techniques for their isolation from various biological materials, which is supervised by the International Society for Extracellular Vesicles (ISEV) (Buzas, 2023).

Although the important role of EVs in biological processes is beyond doubt, numerous studies are being undertaken to fully understand their specific functions in particular physiological and pathological conditions, such as tumorigenesis and inflammatory diseases (Buzas, 2023). The goal of this Research Topic was to present and discuss the recent findings and future perspectives on EV’s biology and functions in modulation of various immune processes with the special focus on tumor-related immunity as well as on allergic and autoimmune responses.

Among other cargoes, EVs convey a variety of bioactive lipids that are encapsulated and/or surface-expressed and play an important but understudied role in EV-induced immunomodulatory effects. Along these lines, Paolino et al. experimentally compared the lipidomes of microvesicle and exosome EV fractions between psoriatic patients and healthy controls, showing the significantly higher concentrations of phospholipids (phosphatidylcholine, phosphatidylethanolamine and phosphatidylglycerol) in psoriasis-related exosomes. Moreover, the Authors investigated the possible changes in the lipid profile of EVs isolated from plasma of psoriatic patients caused by the treatment with monoclonal antibodies against IL-17A, TNFα or IL-12/IL-23p40, and found that therapeutic administration of ustekinumab (a monoclonal antibody against p40, a common subunit of IL-12 and IL-23) restores phosphatidylcholine and phosphatidylethanolamine levels in plasma exosomes to levels detectable in healthy subjects. Thus, it was suggested that phospholipid determination in circulating EVs could serve as a diagnostic determinant and a promising biomarker of drug response in psoriatic patients.

Since EVs can cross anatomical barriers, they are also considered interesting candidates for monitoring processes in healthy and injured immune-privileged organs, such as the brain. Moreover, EVs have been widely studied in the context of intracellular communication between tumors and their microenvironment, promoting an immunosuppressive phenotype. Accordingly, Low et al. summarized the current research data on the impact of EVs on intercellular communication in neoplastic diseases of the central nervous system (CNS) with a special focus on glioblastoma. In most reviewed studies, EVs secreted by glioblastoma cells have been found to promote tumor cell growth and division in an immunosuppressive microenvironment driven by M2 macrophages that provide proangiogenic and promitotic factors. In addition, the Authors highlight the role of direct dysregulation of the activity of particular populations of myeloid cells, T and B lymphocytes, and natural killer cells in the immune suppression induced by tumor-derived EVs. It is worth noting that the blood-CSN barrier itself separates immune cells from the immune-privileged organ, which provides physiological immunotolerance, but also significantly impairs the cellular immune response against tumor cells and infectious viruses.

On the other hand, EVs are extensively investigated to uncover their therapeutic potential. EV-based therapies offer important advantages, consisting on cell-free approaches, that are biocompatible, showing low cytotoxicity and immunogenicity. However, these treatments exhibit disadvantages limiting their use in clinics, such as low stability and delivery efficiency or the uncontrolled specificity of their cargo. In this issue, Liu et al. focused their review article on the role of engineered EVs in the treatment of various inflammatory diseases. In addition to discussing and comparing the cargos of natural and modified EVs, the Authors recapitulate the technics of EV’s modification, applicable routes of their therapeutic delivery and finally the possible targets and outcomes of such therapy in pathologies of the respiratory, digestive, cardiovascular, genitourinary, reproductive, osteoarticular, nervous, immunological and hematological systems.

When considering the therapeutic use of EVs, their bioavailability, which could be limited by the mononuclear phagocyte system (MPS), cannot be overlooked. Namely, studies on the biodistribution of therapeutically administered EVs have shown that they can reach various distant organs, but are detected in the greatest amounts in MPS-enriched tissues of the liver, spleen, kidneys and lungs. These observations suggest that MPS cells rapidly clear exogenous EVs from the circulation, limiting the bioavailability of EV-based therapeutics. Therefore, researchers are now proposing novel strategies to avoid EV removal by MPS cells, as summarized by Cieślik et al. However, Cieślik et al. also point to mononuclear phagocytes as desired EV’s recipients that then can multiply and/or remake EV-delivered signaling to enhance its specificity and enable effective targeting of ultimate acceptor cells. Moreover, the Authors suggest that EVs may produce therapeutic effects by modulating malfunctioning MPS cells in various inflammatory diseases, for example, by restoring dysregulated activity and unbalanced polarization of macrophages.

Such a rapidly growing research field opens countless possibilities for the practical application of new knowledge about EVs, which, however, requires overcoming many limitations and challenges (van Niel et al., 2022), especially when considering EVs as tools for drug delivery (Elsharkasy et al., 2020). This Research Topic provided a platform for organizing the recent findings and future perspectives on EV’s biology and functions in various immune-related processes. We hope that the published results and conclusions will become an impulse for new research discoveries to overcome some of the current drawbacks for EV-based therapies in a way that will determine the future directions of clinical practice.
